# Fucoxanthin Abrogates Ionizing Radiation-Induced Inflammatory Responses by Modulating Sirtuin 1 in Macrophages

**DOI:** 10.3390/md21120635

**Published:** 2023-12-12

**Authors:** Hyunju Kang, Seon-Chil Kim, Youngkee Oh

**Affiliations:** 1 Department of Food and Nutrition, Keimyung University, 1095 Dalgubeol-Daero, Daegu 42601, Republic of Korea; hyunjukang@kmu.ac.kr; 2 Department of Biomedical Engineering, Keimyung University, 1095 Dalgubeol-Daero, Daegu 42601, Republic of Korea; 3 Department of Medical Informatics, School of Medicine, Keimyung University, 1095 Dalgubeol-Daero, Daegu 42601, Republic of Korea; 4 Department of Radiation Oncology, School of Medicine, Keimyung University, 1095 Dalgubeol-Daero, Daegu 42601, Republic of Korea; ykoh@dsmc.or.kr

**Keywords:** fucoxanthin, ionizing radiation, inflammation, sirtuin 1, macrophage

## Abstract

Ionizing radiation (IR) triggers an overproduction of reactive oxygen species (ROS), disrupting the normal function of both immune and metabolic systems, leading to inflammation and metabolic disturbances. To address the pressing requirement for protection against IR, fucoxanthin (FX), a naturally occurring compound extracted from algae, was utilized as an efficient radioprotective agent in macrophages. In this study, we cultured murine RAW 264.7 macrophages and treated them with FX, along with agents influencing the activity of sirtuin 1 (SIRT1) and estrogen receptor α (ERα), to investigate their impact on IR-induced cellular responses. FX significantly attenuated IR-induced upregulation of pro-inflammatory genes (*Il1b*, *Tnf*, and *Ccl2*) and inhibited macrophage polarization toward the pro-inflammatory M1 phenotype. Additionally, FX regulated IR-induced metabolic genes mediating glycolysis and mitochondrial biogenesis. The ability of FX to mitigate IR-induced inflammation and glycolysis was ascribed to the expression and activity of SIRT1 and ERα in macrophages. This study not only uncovers the underlying mechanisms of FX's radioprotective properties but also highlights its potential as a protective agent against the detrimental effects of IR, thus offering new opportunities for enhancing radiation protection in the future.

## 1. Introduction

In the wake of a recent nuclear power plant incident in Japan, there has been a notable surge in the attention paid toward safeguarding against radioactivity in everyday life [1]. Ionizing radiation (IR) is ubiquitous in both natural and anthropogenic settings, spanning from its application in medical diagnostics and treatments to its involvement in nuclear power generation. The growing concern regarding IR arises from its capacity to induce tissue damage, DNA mutations, and various adverse biological effects [2]. In the context of cancer treatment with radiation, DNA damage occurs in both tumor cells and neighboring normal tissues, both directly and indirectly. This damage stems from the generation of reactive oxygen species (ROS) induced by IR, which subsequently triggers inflammation and oxidative stress [3].

Macrophages assume a critical role in the context of IR-induced inflammation, owing to their innate immune response and heightened susceptibility to IR [4]. Upon exposure to IR, macrophages initiate several signaling pathways, releasing inflammatory cytokines and ROS [5], and adopting the M1 phenotype, characterized by classical activation. This inflammatory cascade can contribute to tissue damage, the development of diseases, or the emergence of other complications resulting from IR exposure. Therefore, the modulation of alternative activation pathways in macrophages may represent a prospective therapeutic approach for mitigating IR-induced injuries.

The development of radiation-protective agents has a longstanding history, and presents challenges, primarily stemming from the occurrence of intermediate metabolites that contribute to side effects [6]. As a result, natural substances, including compounds derived from ginseng, aloe, and seaweeds, have been employed as protective agents against IR, owing to their capacity to stimulate the immune system and facilitate cell proliferation [7,8]. Fucoxanthin (FX), a marine carotenoid predominantly found in algae, has recently emerged as a promising candidate for radioprotection due to its robust antioxidant and anti-inflammatory properties [9]. The distinctive structure of FX, characterized by an allenic bond and nine conjugated double bonds, featuring 5,6-monoepoxide and oxygen functional groups, underlies its pharmacological efficacy [10]. Research has demonstrated that FX may ameliorate the adverse effects of IR through its anti-radiation and anti-DNA damage capabilities [11,12]. Furthermore, it has been revealed that the supplementation of FX has no adverse side effects in both in vitro and in vivo systems [13,14,15].

Sirtuin 1 (SIRT1), a nicotinamide adenine dinucleotide (NAD+)-dependent deacetylase, plays a pivotal role in regulating inflammation, oxidative stress, and metabolic disorders associated with excessive alcohol consumption [16,17]. Accumulating evidence suggests that the capacity of FX to mitigate oxidative damage, neural cell death, and diabetic nephropathy is linked to the activation of SIRT1 [18,19]. These findings imply that the radioprotective potential of FX may be intricately linked to the activation of SIRT1 in macrophages. Furthermore, the anti-inflammatory function of SIRT1 can intersect with estrogens [20,21], specifically in the form of ERα, which can induce the M2 phenotype in macrophages [22]. The interplay between SIRT1 and ERα may cooperatively influence the balance between M1 and M2 phenotypes in macrophages exposed to IR. Therefore, it is valuable to examine the roles of SIRT1 and ERα in regulating macrophage function under IR exposure conditions. This study, thus, aimed to investigate the effects of FX on IR-induced inflammatory responses in macrophages, including their polarization, by examining genes associated with inflammation and metabolism. The interaction between SIRT1 and ERα was also discussed to elucidate the underlying mechanisms. This study could lay the groundwork for the development of more effective and natural protective agents against radiation exposure.

## 2. Results

### 2.1. FX Repressed the Expression of IR-Induced Inflammatory Genes in RAW 264.7 Macrophages

**Figure 1:**
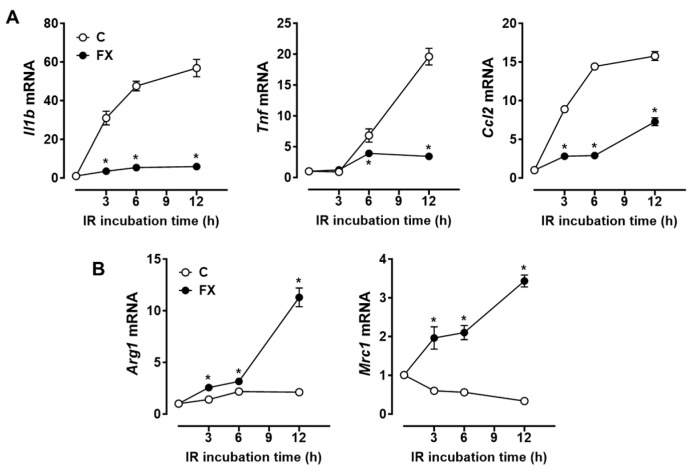
FX affected the expression of inflammatory M1 and anti-inflammatory M2 markers in RAW 264.7 macrophages. Cells were pretreated with 0 (control; C) or 5 µM of FX for 24 h and then stimulated at 6 Gy of IR for 0, 3, 6, and 12 h. (**A**) Expression of M1 markers. (**B**) Expression of M2 markers. No IR control normalized data. Data are shown as mean ± SEM. * *p* < 0.05 vs. control.

We explored whether FX exerts its anti-inflammatory effects on macrophages exposed to IR. Exposure to IR significantly increased the mRNA levels of M1 markers, including interleukin-1β (*Il1b*), tumor necrosis factor α (*Tnf*), and C-C motif chemokine ligand 2 (*Ccl2*). These M1 markers were significantly decreased by FX treatment in RAW 264.7 macrophages (Figure 1A). The findings showed that FX suppressed the expression of IR-induced inflammatory M1 markers, an effect resulting from the polarization of macrophages. Following this, we proceeded to investigate whether the anti-inflammatory properties of FX could be ascribed to its capacity to promote macrophage polarization toward the M2 phenotype. Previous research indicates that a transition in the macrophage population towards the M2 type may confer protection against inflammation associated with IR [23,24]. IR exposure reduced the mRNA levels of M2 markers, including arginase (*Arg1*) and mannose receptor C-type 1 (*Mrc1*); however, FX significantly increased the expression of *Arg1* and *Mrc1* in macrophages (Figure 1B). These findings indicate that FX can suppress the IR-induced inflammatory response in macrophages.

### 2.2. FX Abolished IR-Induced Changes in Metabolic Genes in RAW 264.7 Macrophage

**Figure 2:**
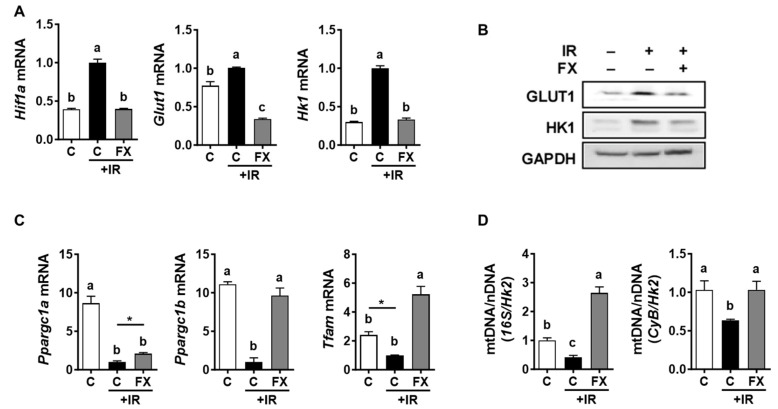
FX affected the genes mediating glycolysis and mitochondria biogenesis in RAW 264.7 macrophages exposed to IR. Cells were pretreated with 5 µM of FX for 24 h and then stimulated with 6 Gy of IR for 12 h. (**A**) Gene expressions of *Hif1*, *Glut1*, and *Hk1* associated with the factors that control energy substrate flow during glycolysis. (**B**) Western blot for protein measurements. (**C**) Corresponding gene expressions of factors involved in mitochondrial biogenesis. (**D**) Mitochondrial DNA copy numbers were determined using *16S* and *CyB* as mitochondrial genes and *Hk2* as a nuclear gene. For Western blot analysis, a representative blot image is shown. Data are shown as means ± SEM. Different letters indicate significant differences (*p* < 0.05).

To examine the alteration of energy metabolism in response to IR, we investigated the effects of both IR and FX on the expressions of genes controlling glycolysis. We focused on hypoxia-induced factor 1α (HIF-1α) and glucose transporter 1 (GLUT1), as GLUT1 is the principal cell membrane glucose transporter in macrophages and is modulated by HIF-1α [25]. We also examined hexokinase 1 (HK1), which facilitates the entry of glucose into macrophages by converting it into glucose-6-phosphate [26]. IR markedly increased the mRNA levels of *Hif1a*, *Glut1*, and *Hk1*; however, these increases were negated by FX in RAW 264.7 macrophages (Figure 2A). Consistently, IR elevated the protein levels of GLUT1 and HK1, but FX counteracted this effect, returning them almost to their original levels (Figure 2B).

Subsequently, our investigation was broadened to evaluate the impact of IR and FX on the expression of genes crucial for mitochondrial biogenesis. IR significantly reduced the mRNA levels of peroxisome proliferator-activated receptor γ coactivator 1α (*Ppargc1a*) and *Ppargc1b*, key activators of mitochondrial biogenesis [27], but this suppression was mitigated by FX (Figure 2C). In line with this, the mRNA level of transcription factor A (*Tfam*) was decreased by IR, but FX reversed this effect. In a parallel manner, the ratio of mitochondrial to genomic DNA, serving as a surrogate marker for the quantity of mitochondrial copies, displayed a notable reduction following exposure to IR. Nonetheless, this decline was effectively mitigated by FX, as illustrated in Figure 2D. These outcomes underscore the potential of FX to counteract the adverse consequences of IR on mitochondrial biogenesis.

### 2.3. FX Abrogated the IR-Induced Decrease in SIRT1 and ERα Expression in RAW 264.7 Macrophages

**Figure 3:**
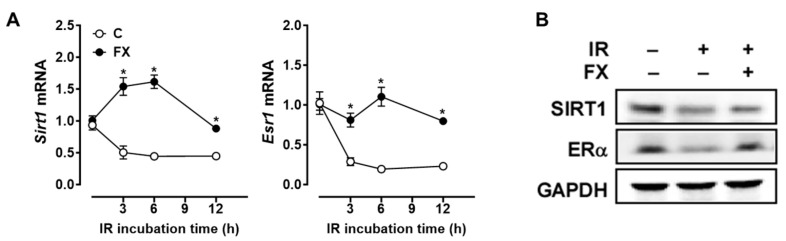
FX restored the IR-induced decrease in SIRT1 and ERα expression in RAW 264.7 macrophages. (**A**) Cells were pretreated with 5 µM of FX for 24 h and then stimulated with 6 Gy of IR for 0, 3, 6, and 12 h for gene analysis. (**B**) Cells were pretreated with 5 µM of FX for 24 h and then stimulated at 6 Gy of IR for 12 h for protein measurements. For the Western blot analysis, a representative blot image is shown. No IR control normalized data. * *p* < 0.05 vs. control.

In light of the downregulation of SIRT1 caused by IR [28], our investigation aimed to explore whether FX could counter the inhibitory effect of IR on the SIRT1 expression in macrophages. Consistently, IR reduced *Sirt1* mRNA levels, but FX mitigated this decrease in RAW 264.7 macrophages (Figure 3A). Specifically, the expression of *Sirt1* decreased in macrophages upon stimulation with 6 Gy of IR for 3 h and then maintained the lower level until 12 h. The supplementation of FX, however, elevated the expression of *Sirt1* for 6 h, followed by a decrease until 12 h. Additionally, evidence suggests that the anti-inflammatory role of SIRT1 is synergistically supported by estrogens [21,29]. Given that estrogens can act through ERα to promote the M2 phenotype [22], the interaction between SIRT1 and ERα may collaboratively influence the balance between M1 and M2 phenotypes in macrophages exposed to IR. Thus, we measured the mRNA levels of *Esr1* (the gene encoding ERα) following IR exposure. IR significantly reduced *Esr1* mRNA levels, but this was restored by FX (Figure 3A). Furthermore, the protein levels of SIRT1 and ERα, which were diminished by IR, were restored by FX in RAW 264.7 macrophages (Figure 3B), indicating that FX counteracts the IR-induced reduction in both SIRT1 and ERα expression.

### 2.4. Alteration of SIRT1 Activity Affected Expression of Pro-Inflammatory Genes in IR-Stimulated Macrophages

**Figure 4:**
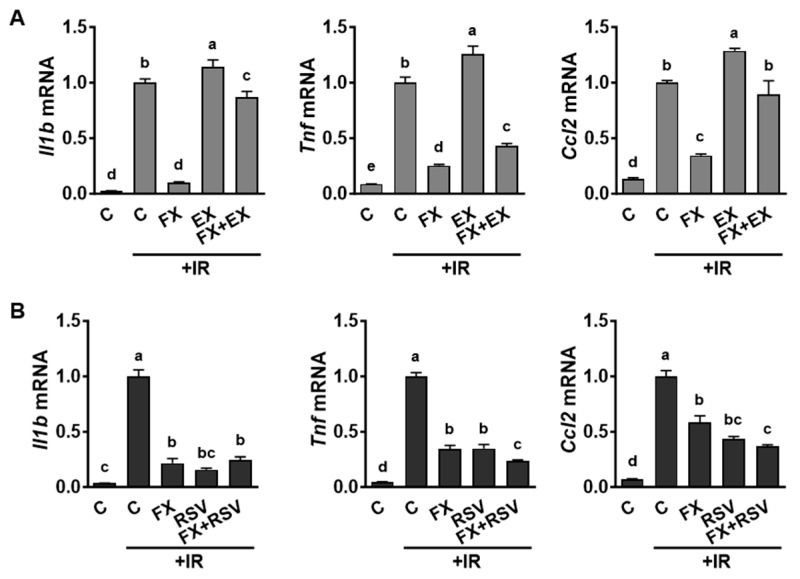
The activity of SIRT1 influenced the expression of pro-inflammatory genes in IR-stimulated RAW 264.7 macrophages. (**A**) Cells were pretreated with 5 µM of FX and 15 µM of EX-527 (EX; a SIRT1 inhibitor) for 24 h, followed by stimulation with 6 Gy of IR for 12 h for gene analysis. (**B**) Cells were pretreated with 5 µM of FX and 20 µM of resveratrol (RSV, a SIRT1 activator) for 24 h and then stimulated with 6 Gy of IR for 12 h for gene analysis. Data are shown as means ± SEM. Different letters indicate significant differences (*p* < 0.05).

Considering the well-established anti-inflammatory attributes of SIRT1 [30], our objective was to elucidate its involvement in the regulation of pro-inflammatory gene expression in macrophages exposed to IR. In order to scrutinize the impact of SIRT1 activity, we utilized EX-527 as a SIRT1 inhibitor and resveratrol as a natural SIRT1 activator, respectively. The IR-induced upregulation of pro-inflammatory genes (*Il1b*, *Tnf*, and *Ccl2*) was further amplified by the addition of the SIRT1 inhibitor, EX-527 (Figure 4A). Conversely, this upregulation was mitigated by resveratrol, a SIRT1 activator (Figure 4B), with a notable additive effect in reducing *Tnf* expression. These results align with previous studies, which have shown that reduced SIRT1 expression is associated with the upregulation of inflammatory genes and an enhanced inflammatory response in various cell types and tissues [31]. The results indicate that FX’s capacity to inhibit the IR-induced upregulation of pro-inflammatory genes can be attributed to the activation of SIRT1, thus affirming the pivotal role of SIRT1 in IR-activated macrophages.

### 2.5. Alteration of ERα Activity Affected the Expression of SIRT1 and Pro-Inflammatory Genes in IR-Stimulated Macrophages

**Figure 5:**
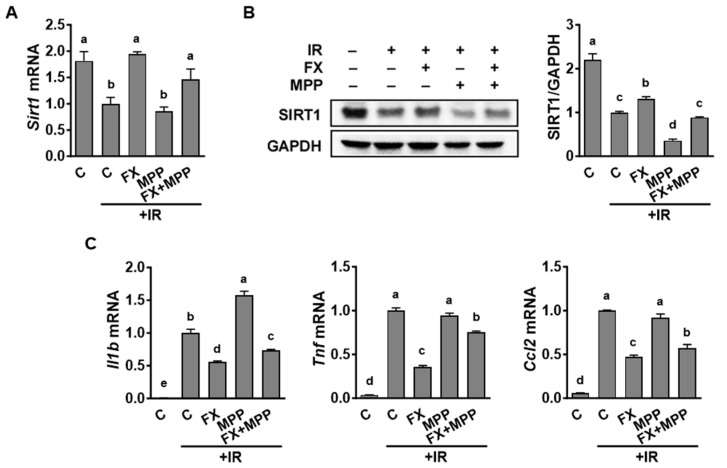
Inhibition of ERα activity repressed the expression of SIRT1 but enhanced the expression of pro-inflammatory genes in IR-stimulated RAW 264.7 macrophages. (**A**,**C**) Cells were pretreated with 5 µM of FX and 100 nM of methyl-piperidino-pyrazole (MPP; an ERα inhibitor) for 24 h and were then stimulated with 6 Gy of IR for 12 h for gene analysis. (**B**) Western blotting was used for protein measurements. For the Western blot analysis, a representative blot image is shown, and a densitometry analysis was conducted using GAPDH for normalization. Data are shown as means ± SEM. Different letters show significant differences (*p* < 0.05).

To delve deeper into the interplay between ERα and SIRT1, we considered the impact of ERα on SIRT1 expression and the modulation of pro-inflammatory gene expression in macrophages in the context of IR stimulation. RAW 264.7 macrophages were treated with either an ERα-targeted inhibitor or an activator and then exposed to IR. The treatment of IR-stimulated macrophages with MPP, a specific ERα inhibitor, did not significantly affect *Sirt1* mRNA levels (Figure 5A) but markedly reduced SIRT1 protein levels in the absence of the FX supplement (Figure 5B). Regarding pro-inflammatory M1 markers, treatment with MPP in IR-stimulated macrophages significantly elevated the mRNA expression levels of *Il1b*. However, MPP did not substantially affect the IR-induced upregulation of *Tnf* and *Ccl2* in macrophages (Figure 5C). Notably, the levels of M1 markers that had been reduced by FX supplementation were significantly increased upon MPP treatment.

**Figure 6:**
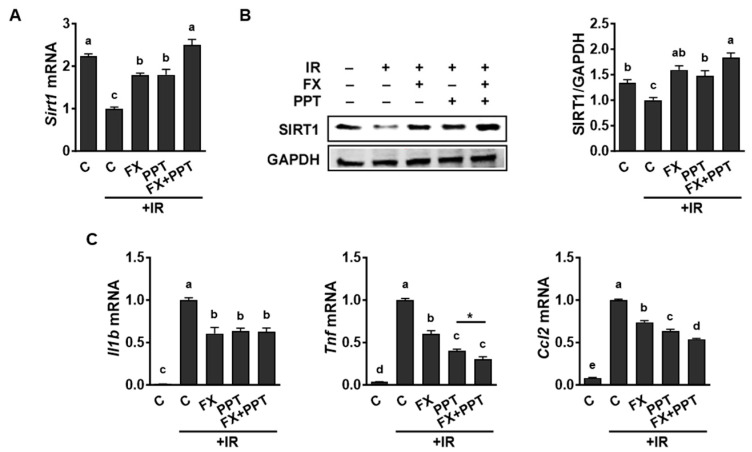
Activation of ERα activity enhanced the expression of SIRT1 but repressed the expression of pro-inflammatory genes in IR-stimulated RAW 264.7 macrophages. (**A**,**C**) Cells pretreated with 5 µM of FX and 10 nM of 4,4′,4″-(4-propyl-[1H]-pyrazole-1,3,5-triyl) trisphenol (PPT, an ERα activator) for 24 h followed by stimulations with 6 Gy of IR for 12 h for gene analysis. (**B**) Western blot was used for protein measurements. For the Western blot analysis, a representative blot image is shown, and a densitometry analysis was conducted using GAPDH for normalization. Data are shown as means ± SEM. Different letters show significant differences (*p* < 0.05).

Conversely, the treatment with PPT, an ERα-specific agonist, led to a considerable increase in SIRT1 expression in both mRNA (Figure 6A) and protein (Figure 6B) levels in IR-stimulated macrophages. Furthermore, treatment with PPT significantly diminished the IR-induced increase in mRNA levels of *Il1b*, *Tnf*, and *Ccl2*. When PPT was combined with FX, there was an additional reduction in *Tnf* and *Ccl2* mRNA levels in IR-stimulated macrophages (Figure 6C). The results demonstrate that the inhibition of ERα activity suppresses SIRT1 expression and concurrently increases the expression of pro-inflammatory genes in IR-stimulated macrophages. Conversely, the activation of ERα activity yields contrasting effects, signifying a functional interplay between SIRT1 and ERα in the regulation of IR-induced inflammatory responses in macrophages.

## 3. Discussion

Macrophages can assume a prominent role in orchestrating the inflammatory response to external stimuli, such as IR. One distinctive characteristic of the macrophage response is their polarization toward either the M1 or M2 phenotype, depending on the nature of the stimulus. This polarization is discerned through the heightened expression of M1 or M2 markers, as appropriate [32,33]. We undertook an examination of the inhibitory effects of FX on the IR-induced inflammatory response in macrophages, as a pivotal step in the quest to formulate natural and efficacious agents against IR while minimizing potential side effects. Our experimental design closely mirrored the conditions typically employed in cancer treatment within medical facilities, with a specific focus on elucidating the direct radioprotective roles of macrophages, which play a pivotal role in inflammatory responses [34]. FX was chosen as a candidate agent on the basis of its well-established safety profile and demonstrated effectiveness, as evidenced by prior investigations [13,14,15]. Upon exposure to ionizing radiation, the absorbed energy triggers cellular ionization [35], subsequently leading to the breakdown of water into reactive oxygen species and DNA mutations [8]. This radiation-induced ionization can induce a cascade of biochemical alterations, cellular anomalies, and biological damage [36]. The effective regulation of ROS production and clearance, along with the attenuation of inflammation, emerge as critical considerations in the development of radioprotective agents. The outcomes of our study unequivocally demonstrated that FX possessed the capacity to mitigate IR-induced inflammatory responses in RAW 264.7 macrophages, inducing polarization toward the M2 phenotype. Aligning seamlessly with the existing literature, the robust anti-inflammatory properties exhibited by FX were ascribed to its unique structure, which scavenges ROS generated upon IR exposure [37]. The repression of pro-inflammatory M1 markers could have far-reaching clinical implications, particularly in conditions exacerbated by IR-induced inflammation, such as radiation-induced fibrosis or cancer.

Research findings have illuminated the close interplay between energy metabolism and immune functions, with a particular focus on the regulation of macrophage polarization. This dynamic interplay propels macrophages to transition between the M1 and M2 phenotypes in response to extracellular signals [32]. The polarization of macrophages to the M2 phenotype by FX successfully abolished the metabolic changes in macrophages following IR exposure. IR-stimulated macrophages may require additional cellular energy to convert H_2_O into ROS and to facilitate an inflammatory microenvironment, which could favor glycolysis over normal oxidative phosphorylation through the TCA cycle [38]. This observation suggests that FX holds promise as a metabolic modulator, mitigating IR-induced metabolic changes that may result in cellular dysfunction or contribute to pathological states. For suppressing IR-induced inflammation and glycolysis in macrophages, the regulation of free electrons and the electron transport chain is crucial [39]. This phenomenon arises due to the fact that IR can lead to the accumulation of excessive electrons, thereby fostering an oxidative microenvironment and triggering the activation of genes associated with inflammation and glycolysis in macrophages [40]. Moreover, the metabolic transition towards glycolysis appears to be intricately linked to the redox equilibrium between NAD+ and NADH, which, in turn, is intimately tied to the overall electron balance within the cell [41]. The efficacy of FX in regulating these electron-related processes may be attributed to its distinctive chemical structure, characterized by a distinctive allenic bond and nine conjugated double bonds [10].

SIRT1 is widely recognized as an inhibitor for the generation of pro-inflammatory cytokines and the accumulation of ROS in macrophages [42]. It acts to suppress the M1 macrophage phenotype while concurrently fostering the anti-inflammatory M2 phenotype through the activation of distinct signaling pathways [43]. Notably, FX reversed the IR-induced reduction in activities and expressions of both SIRT1 and ERα, positioning it as a promising natural agent for activating these key regulators. Prior research has established that ERα can engage with estrogen response elements (EREs) within the promoter region of the SIRT1 gene, resulting in elevated SIRT1 expression [44]. SIRT1 is renowned for its contributions to stress resistance, metabolic regulation, and extension of lifespan, while ERα is implicated in diverse cellular processes, encompassing growth and the modulation of inflammatory responses [45]. Previous investigations have emphasized the potential crosstalk between SIRT1 and ERα in regulating macrophage function, especially in stressful situations such as exposure to IR [46]. As a nuclear hormone receptor, ERα targets genes associated with inflammation, energy metabolism, and cellular stress responses, akin to SIRT1. Research has demonstrated that SIRT1 activation results in elevated ERα protein levels, whereas its inhibition diminishes these levels [44]. Additionally, SIRT1 can modulate ERα activity by deacetylating it [29]. ERα, in turn, may reciprocally influence SIRT1 expression or activity, as ERα activation can upregulate the SIRT1 expression and reduce inflammation by enhancing the SIRT1-mediated deacetylation of inflammatory transcription factors such as NF-κB [31]. Recent research has further accentuated the influence of ERα on macrophage characteristics, offering insights into its intricate interplay with SIRT1 [47]. The involvement of ERα in steering macrophage polarization, a process pertinent to the development of numerous inflammatory diseases, has garnered growing interest [48]. Consequently, our findings suggest that FX’s efficacy in attenuating the IR-induced upregulation of pro-inflammatory and glycolytic genes in macrophages is linked to the interplay between SIRT1 and ERα. Specifically, FX appears to shift macrophage polarization from an M1 to an M2 phenotype, likely through the synergistic action of SIRT1 and ERα. Taken together, FX exerts its anti-inflammatory effects via the SIRT1 signaling pathway, which governs inflammation, glycolysis, and macrophage polarization and is further modulated by ERα. These findings suggest that FX counteracts the IR-induced reduction in SIRT1 and ERα expression, implying a protective interplay between these two genes against IR-induced risks.

Although our findings offer robust preliminary evidence for the radioprotective abilities of FX, a comprehensive understanding of the underlying molecular mechanisms remains to be elucidated. Subsequent research endeavors should prioritize the exploration of the interplay among FX, SIRT1, and ERα signaling pathways. Comprehending these intricate interactions is imperative for the development of natural and efficacious radioprotective agents capable of ameliorating the detrimental consequences of exposure to ionizing radiation.

## 4. Methods and Materials

### 4.1. Cell Cultures and Treatments

Murine RAW 264.7 macrophages (ATCC, Manassas, VA, USA) were maintained in RPMI 1640 media (Welgene Inc. Daegu, Republic of Korea), containing 10% fetal bovine serum, 100 U/mL penicillin, 100 μg/mL streptomycin, 1× vitamins, and 2 mmol/L L-glutamine, as previously described [49]. Cells were maintained at 37 °C in a 5% CO₂ atmosphere. RAW 264.7 cells were plated in 12-well plates at 2 × 10^5^ cells/well for gene analysis and 4 × 10^5^ cells/well for protein analysis. FX was acquired from Sigma-Aldrich (St. Louis, MO, USA), along with EX-527 (EX), a SIRT1 inhibitor, and resveratrol (RSV), a SIRT1 activator. FX, EX, and RSV were used in the experiments at the final concentrations of 5 μM, 15 μM, and 20 μM, respectively. 4,4′,4″-(4-propyl-[1H]-pyrazole-1,3,5-triyl) trisphenol (PPT), an agonist of estrogen receptor α (ERα), and methyl-piperidino-pyrazole dihydrochloride (MPP), an ERα-specific inhibitor, were purchased from Tocris Bioscience (Bristol, UK). The final concentrations for PPT and MPP in the experiments were 10 nM and 100 nM, respectively.

### 4.2. RNA Extraction and Quantitative Real-Time PCR (qRT-PCR)

Total RNA extraction from the cells was carried out using NucleoZOL reagent (MA-CHEREY-NAGEL, Düren, Germany), following the manufacturer’s recommended protocols. Subsequently, reverse transcription for cDNA synthesis was performed using the Revertra Ace qPCR RT master mix (Toyobo, Osaka, Japan). The quantitative real-time polymerase chain reaction (qRT-PCR) was conducted using a Bio-Rad CFX Duet Real-Time PCR System (Bio-Rad, Hercules, CA, USA). These procedures were independently replicated in three separate experiments. Primer design was accomplished with the assistance of Beacon Designer 7 software (PREMIER Biosoft International, Palo Alto, CA, USA), utilizing information sourced from the GenBank database, as previously mentioned [50,51].

### 4.3. Western Blot Analysis

For whole-cell lysate preparation and Western blot analysis, methods as previously detailed were employed [52,53]. Antibodies for SIRT1, ERα, hexokinase 1 (HK1), and glyceraldehyde 3-phosphate dehydrogenase (GAPDH) were purchased from Santa Cruz Biotechnology (Santa Cruz, CA, USA). Antibodies for glucose transporter 1 (GLUT1) were purchased from Cell Signaling Technology (Danvers, MA, USA). GAPDH was used as a loading control to normalize the data. The blots were visualized using ChemiDoc (Bio-Rad) or the iBright Imaging System (Invitrogen, Carlsbad, CA, USA). Independent experiments were performed at least three times.

### 4.4. Mitochondrial DNA Copy Number

Total DNA was isolated from RAW 264.7 macrophages treated with 6 Gy of IR in the absence or presence of 5 μM of FX for 12 h using a Clear-S™ Quick DNA Extraction Kit (Invirustech, Gwangju, Republic of Korea). The mitochondrial DNA copy number was determined using 16S ribosomal RNA (*16S*) and cytochrome b (*CyB*) as mitochondrial genes and hexokinase 2 (*Hk2*) as a nuclear gene. At least three independent experiments were conducted.

### 4.5. Irradiation

**Figure 7:**
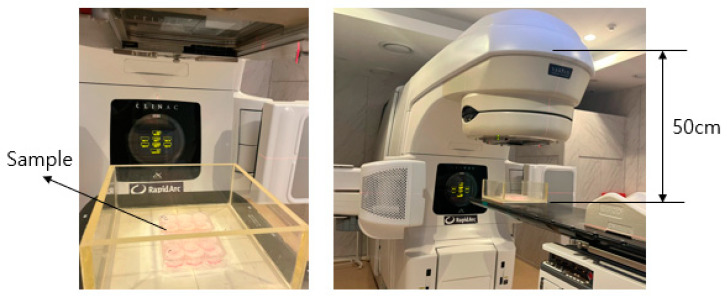
Radiated sample and irradiation method setup.

In this study, an X-ray irradiator, specifically the Varian Linac iX (New Bedford, MA, USA), was employed as the radiation source for cancer treatment. The irradiator had a maximum output of 600 MU/min, and the samples were irradiated at a distance of 50 cm, delivering a dose of 6 Gy per irradiation, as visually represented in Figure 7 [54]. The choice of a 50 cm distance aimed to guarantee a uniform distribution of the radiation dose across the samples, mimicking the distance employed in human treatment. The selected 6 Gy dose aligned with the study’s objective of rapidly identifying severely irradiated individuals in mass-casualty and population-monitoring scenarios, which indicates a consideration for clinical relevance [55]. In previous cytotoxicity tests using MTT assays with varying doses of gamma radiation (4–10 Gy) on RAW 264.7 macrophage cells, cell viability was not significantly affected at doses below 10 Gy [56,57]. Based on these results, a dose of 6 Gy was selected in subsequent experiments to ensure a consistent response in macrophages while avoiding significant impacts on cell viability.

### 4.6. Statistical Analysis

Statistical analyses were conducted to assess significant differences between groups using GraphPad Prism 7.0 software (GraphPad Software, La Jolla, CA, USA), in accordance with previously described procedures [58,59]. The statistical analysis employed in this study encompassed one-way analysis of variance (ANOVA) alongside the Newman–Keuls post hoc test, or unpaired t-tests. Statistical significance was established when the *p*-value was less than 0.05. The data were presented as means ± SEM.

## 5. Conclusions

This study marks a significant stride in the pursuit of radiation-protective agents for application within medical settings. We have contributed novel insights into the diverse protective functions of FX against the repercussions of IR on macrophages in tissues, underscoring its potential as a therapeutic agent. FX regulated the IR-induced expression of pro-inflammatory genes, macrophage polarization toward the M1 phenotype, and alterations in the genes associated with glycolysis and mitochondrial biogenesis. The study revealed that FX’s anti-inflammatory effects in IR-stimulated macrophages are mediated through SIRT1 activation. Additionally, FX appeared to modulate a complex network of signaling pathways involving SIRT1 and ERα, thus suggesting synergistic roles in anti-inflammatory responses. Hence, these findings posit that FX harbors the potential to function as a radiation-protective agent on macrophages.

## Data Availability

Data are contained within the article.
